# Langerhans Cells in Sentinel Lymph Nodes from Melanoma Patients

**DOI:** 10.3390/cancers16101890

**Published:** 2024-05-16

**Authors:** Gianni Gerlini, Pietro Susini, Serena Sestini, Paola Brandani, Vanni Giannotti, Lorenzo Borgognoni

**Affiliations:** 1 Plastic and Reconstructive Surgery Unit, Regional Melanoma Referral Center and Melanoma & Skin Cancer Unit, Santa Maria Annunziata Hospital, 50012 Florence, Italy; serena.sestini@uslcentro.toscana.it (S.S.); paola.brandani@uslcentro.toscana.it (P.B.); vanni.giannotti@uslcentro.toscana.it (V.G.); lorenzo.borgognoni@uslcentro.toscana.it (L.B.); 2 Plastic Surgery Unit, Department of Medicine, Surgery and Neuroscience, University of Siena, 53100 Siena, Italy; susinipietro@gmail.com

**Keywords:** Langerhans cells and melanoma, Langerhans cells and sentinel lymph node, Langerhans cells and metastasis, Langerhans cells and immunotherapy

## Abstract

**Simple Summary:**

Melanoma, the deadliest skin cancer, is a focus of research worldwide. This malignancy has been demonstrated to evade the immune system through several escape mechanisms. Among these, melanoma-related Dendritic Cells (DCs) alterations, particularly within the Sentinel Lymph Node (SLN), seem to play a key role. In SLNs, melanoma-related factors create a tumor microenvironment capable of impairing the immune response, inducing tolerance to tumor antigens and thus favoring SLN early metastases. The present literature review describes the interactions between Langerhans Cell (LC), a particular DC subset, and melanoma, suggesting a new potential therapeutic target.

**Abstract:**

Background. Langerhans cells (LCs) are professional Dendritic Cells (DCs) involved in immunoregulatory functions. At the skin level, LCs are immature. In response to tissue injuries, they migrate to regional Lymph Nodes (LNs), reaching a full maturation state. Then, they become effective antigen-presenting cells (APCs) that induce anti-cancer responses. Notably, melanoma patients present several DC alterations in the Sentinel Lymph Node (SLN), where primary antitumoral immunity is generated. LCs are the most represented DCs subset in melanoma SLNs and are expected to play a key role in the anti-melanoma response. With this paper, we aim to review the current knowledge and future perspectives regarding LCs and melanoma. Methods. A systematic review was carried out according to the PRISMA statement using the PubMed (MEDLINE) library from January 2004 to January 2024, searching for original studies discussing LC in melanoma. Results. The final synthesis included 15 articles. Several papers revealed significant LCs–melanoma interactions. Conclusions. Melanoma immune escape mechanisms include SLN LC alterations, favoring LN metastasis arrival/homing and melanoma proliferation. The SLN LCs of melanoma patients are defective but not irreversibly, and their function may be restored by appropriate stimuli. Thus, LCs represent a promising target for future immunotherapeutic strategies and cancer vaccines.

## 1. Introduction

Dendritic Cells (DCs) are professional Antigen-Presenting Cells (APCs) involved in maintaining the balance between immunity and tolerance [1]. Among DC subsets, Langerhans cells (LCs) reside within the epidermis and mucosae and represent an important first line of defense, patrolling in search of pathogen invasion and priming immune responses [1,2]. LCs are in the immature state within the epithelia [3]. In response to tissue injuries, they migrate to regional Lymph Nodes (LNs) where, after reaching a full maturation state, they acquire the ability to activate T lymphocytes [3]. LCs are strategically localized above the basal layer of epithelial cells, representing the first DCs to deal with skin cancer, including melanoma. They are hypothesized to play a key role in the generation of antitumor immune responses [4]. However, the specific immune function of LCs is controversial and a matter of debate [5].

The historical dogma about LCs, “immature in the periphery and mature in LNs”, has been challenged by the recent identification of immature LCs in LNs, possibly responsible for tolerance towards self-antigens [6]. In addition, LCs stimulated by mature interferon-gamma transiently upregulate the enzyme indoleamine 2,3-dioxygenase (IDO). The latter exerts strong tolerogenic action, promoting peripheral tolerance [7,8], including tumor tolerance [9]. Therefore, IDO expression by LCs could be responsible for regulatory/inhibitory functions of the subset, potentially relevant in melanoma [7]. Moreover, melanoma cells have been related to DC alterations within the Sentinel Lymph Node (SLN), the first node draining from a tumor site, where primary immune responses to tumor antigens are expected to occur [10].

Given these considerations, resident epithelial LCs could become an innovative target for cancer immunotherapy [4,11]. Furthermore, the role of LCs in the immune system both peripherally and at the SLN, as well as the relationship between LC maturation and IDO expression, are hot topics and deserve further investigation. With the present review, we focus on the role of LCs in melanoma and possible therapeutic strategies based on LCs. Sporadic, brief reports on other DC subsets are also mentioned.

## 2. Materials and Methods

### 2.1. Data Sources and Search Strategy

A systematic review of the literature was carried out according to the PRISMA statement [12] for Systematic Reviews by searching the PubMed (MEDLINE) library from January 2004 to January 2024 using the terms ‘‘(Langerhans cells and melanoma) OR (Langerhans cells and sentinel lymph node) OR (Langerhans cells and metastasis) OR (Langerhans cells and immunotherapy)”. This systematic review was registered in the International Prospective Register of Systematic Reviews (PROSPERO) (ID: CRD42024531399).

### 2.2. Study Selection

Inclusion criteria consisted of original studies conducted on humans (observational study or randomized controlled trial) discussing the role of LC in melanoma. Exclusion criteria were animal studies, reviews and meta-analyses, books and documents, case reports, and papers not written in English. Studies reporting incomplete, inconclusive, or descriptive results were also excluded. Publications were screened based on titles and abstracts to determine whether they met the selection criteria or not. When it was difficult to evaluate the eligibility of the papers based on the title and abstract alone, the full text was reviewed and compared to the selection criteria. The bibliographical references of the most relevant articles were also evaluated. After the first screening by title and abstract, the selected articles were subjected to full-text review and tested with the selection criteria. Following study selection, data extraction, and critical appraisal, the collected data were brought to the attention of the senior author (LB) for any disagreement resolution and final approval. Through this method, the papers were re-examined and finally included in the review.

## 3. Results

**Figure 1:**
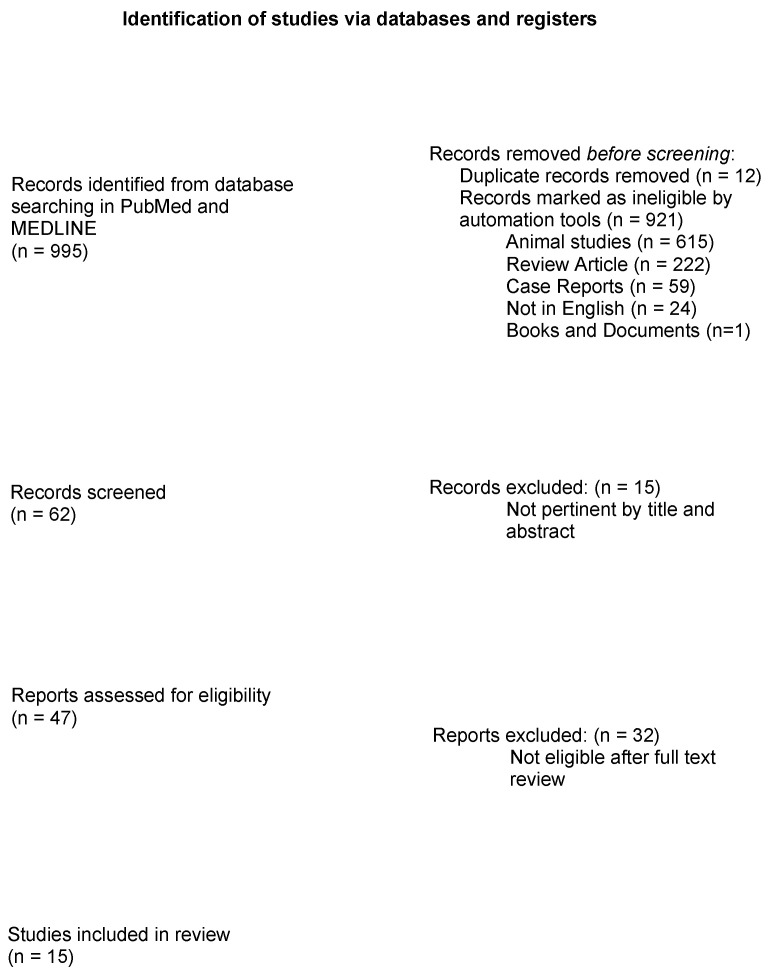
PRISMA 2020 Flow diagram summarizing research results.

With the established keywords, the primary research found a total of 995 articles (Figure 1). These were compared to selection criteria. Through the use of PubMed’s automatic search tools, 615 animal studies, 222 reviews and meta-analyses, 59 case reports, 24 articles not written in English, and one book and document were excluded. Twelve duplicates were also excluded. A total of 75 remaining articles were assessed for relevance based on their title and abstract; as a result, 47 potentially eligible original articles were selected and fully reviewed. Of these, 32 articles not relevant to the scope of this paper were excluded. Finally, 15 articles met the selection criteria and were included in this review (Table 1).

## 4. Discussion

### 4.1. Melanoma and Sentinel Lymph Node

Melanoma is the deadliest skin cancer, and its incidence is rapidly increasing, being six times higher than 40 years ago [27]. Tumor cells, particularly melanoma, can evade the immune system through several escape mechanisms [5]. Among these, melanoma has been demonstrated to alter DC functions, particularly within the SLN, where it can induce tolerance to tumor antigens, favoring SLN early metastases [28,29]. In a routine clinical setting, SLN biopsy is of outstanding importance: it is a surgical procedure aimed at identifying clinically occult regional metastases of the LNs, providing an excellent opportunity to study early immune responses to cancer. It is also the most accurate method of risk assessment and correct staging for patients with invasive melanoma [29,30].

It has been demonstrated that SLNs in cancer patients, but especially melanoma patients, have altered functions [10,28]. Moreover, melanoma cells have been shown to release immunosuppressive cytokines, including IL-10 and TGF-beta. These could affect SLN DCs negatively, promoting an immunosuppressive microenvironment [25,31,32]. Notably, melanoma SLN DCs exhibit several alterations compared to healthy LN DCs, ranging from histopathological aberrations to immunophenotype and functional defects [10,28,33,34]. Accordingly, melanoma-induced immunosuppressive microenvironment may favor SLN early metastases, a crucial step in tumor progression [35].

Recently, new immune and targeted therapies have become established in managing advanced melanoma, significantly improving survival rates. Immune checkpoint inhibitors, including anti-CTLA4 and anti-PD-1, restore the immune system by “removing the brake”, promoting CD8-positive T-cells anti-cancer activity, enabling tumor regression and long-term cancer control in up to 50% of patients [36,37,38]. Indeed, in this intriguing era of immunotherapy for advanced melanoma, the detailed analysis of resident LCs and migrated LCs in SLN may provide important insights into designing new immunotherapeutic strategies.

### 4.2. Peripheral Resident Langerhans Cells

The skin hosts epidermal LCs and dermal DCs subsets and a crowded lymphatic network to facilitate the capture and transport of antigens (including tumoral antigens) to regional draining LNs. DCs, and particularly LCs, are considered to be initiators of an efficient antitumor immune response, thus representing a topic of interest for tumor research [39,40,41]. Peripheral resident LCs are in the immature state within the epithelia, which is characterized by the expression of surface markers CD1a and Langerin [3]. In response to tissue injuries or proinflammatory cytokines, they migrate to regional lymph nodes, reaching a full maturation state, upregulating class I-II MHC molecules, CD80/CD86 co-stimulation and CD83 maturation markers [3,23]. This immunophenotype is crucial for LCs to present antigens to naïve T-cells and, consequently, support antitumor immunity [3,23].

### 4.3. Langerhans Cells in Sentinel Lymph Node

LCs are the most represented DC subset in melanoma SLNs [42]. An important contribution to the current knowledge on DCs in SLNs comes from a Dutch group that developed a method to obtain viable cells from melanoma-draining SLNs without compromising routine diagnostic procedures [43,44], offering the possibility to analyze SLN DCs in patients with melanoma. In particular, melanoma appears to have an effect on SLN DCs, including LCs, compromising their immunoregulatory functions. Hot topics and controversies regarding the functions and roles of LCs in melanoma are described below.

#### 4.3.1. Langerhans Cells in Melanoma Sentinel Lymph Node Are Functionally Defective

LCs have been studied in both metastatic (melanoma-positive) and metastasis-free (melanoma-negative) SNLs. Van de Ven et al. [21] demonstrated that melanoma-negative SLN LCs are phenotypically mature. Surprisingly, the authors revealed that these are functionally defective, release lower levels of inflammatory cytokines, less T-cell priming and IFNγ induction [21]. Moreover, Romoli et al. [15] studied the antigen-presenting machinery (APM) in SLN LCs of patients with or without melanoma. Interestingly, HLA-class I APM component levels (Delta, LMP-7/10, TAP-1, Calnexin, Tapasin, β2-microglobulin, and HLA-A,B,C) were lower in immature epidermal LCs compared to melanoma-positive SLN LCs, and significantly increased after LCs maturation. Overall, melanoma seems to promote functional deficits in LCs, impairing their immune-regulatory functions and possibly promoting tumor tolerance.

#### 4.3.2. Langerhans Cells in Melanoma Sentinel Lymph Node Are Immature

Complementing these studies, Gerlini et al. [20] compared melanoma-negative and melanoma-positive SLNs to freshly isolated and epidermal explant–migrated LCs as models for immature and mature LCs, respectively. The Authors investigated 16 melanoma-positive SLNs (average Breslow thickness 3.21 mm; the interval from a melanoma excision of 0–39 days, mean 10 days) and 27 melanoma-negative SLNs (average Breslow thickness 2.43 mm, interval from melanoma excision 0–56 days, mean 16 days) [20]. They revealed that both melanoma-negative and positive SLN LCs expressed lower levels of CD83, CD80, CD86, and HLA-DR (maturation markers) compared to epidermal explant–migrated (mature) LCs. By contrast, marker expression was similar to freshly isolated LCs, suggesting that SLN LC features are similar to immature LCs, both phenotypically and functionally [20]. Indeed, they preserve the ability to uptake and process exogenous antigens, a peculiar function of immature DCs, which is then lost by mature DCs [1]. Such evidence is contradictory when compared to the study of Van de Ven et al. [21], who showed that LCs are functionally defective but phenotypically mature, thus opening the debate.

Notably, melanoma-negative SLN LCs expression of CD80 and CD86 co-stimulatory molecules was similar to that reported by van de Ven et al. [21]. However, the CD83 maturation marker was expressed in a considerably lower percentage (16.68 ± 7.73% vs. 45–50%), suggesting that melanoma patients’ LCs migrate from the skin to SLNs, preserving immature phenotype, possibly tolerating melanoma-associated antigens and promoting cancer spread [45]. Alternatively, the immature LC immunophenotype could be secondary to the differences in intervals between primary melanoma excision and SLN biopsy (16 days vs. 44 days for Gerlini et al. and van de Ven et al., respectively [20,21]). Indeed, a shorter interval could explain the preservation of melanoma-induced suppressive effects on SLNs. Overall, SLN LCs display an immature immunophenotype characterized by low DC maturation marker CD83 expression and low co-stimulatory molecules [20,21]. Although these findings may indicate a steady-state condition [46], the data strongly suggest a melanoma-related effect. Specifically, these defects may be responsible for LCs’ inability to present tumor antigens to cytotoxic CD8+ T cells in SLN, which has a significant impact on anti-melanoma immunity.

#### 4.3.3. Langerhans Cells Activation State in Melanoma Sentinel Lymph Node Is Affected by Breslow Thickness and Excision Interval

In agreement with the hypothesis that the SLN LC maturation state is impaired by melanoma, the frequency of SLN mature LCs could indirectly correlate to Breslow’s thickness of the primary lesions and directly correlate to the mean interval from melanoma excision. The latter could be relevant since melanoma-induced LC immunosuppression is expected to decrease over time following primary cancer excision. In particular, van den Hout et al. [19] showed that melanoma-negative SLN biopsy performed between 12 and 94 days from primary tumor excision hosted skin-migrated LCs with a semimature phenotype characterized by CD83 maturation marker expression in 50% compared to 17% as for Gerlini et al. [20]. However, the Authors argue that there might be significant differences in the studio populations. Specifically, the van den Hout et al. [19] group reflects a scenario characterized by a predominance of tumor-negative SLNs and relatively long intervals between primary melanoma excision and SLN biopsy with a 44-day mean interval versus 16 days in ours [19,20]. Notably, they reported a minimal, yet not statistically significant, increase in LCs CD83+ maturation marker expression for longer tumor excision—SLN biopsy intervals.

In addition, the mean Breslow thickness in the SLN-negative patients group studied by Gerlini was 2.43 mm vs. 1.5 mm in the group studied by van den Hout [19,20]. Consistently, they suggest that LC CD83 expression is inversely related to Breslow thickness. Moreover, the authors questioned whether the melanoma-positive SLN LCs group by Gerlini expressed lower levels of CD83 due to the higher Breslow thickness (mean 3.21 mm) rather than melanoma in the SLN [19,20]. Combining the data, the observed difference in LC activation state (CD83, CD80, CD86 expression) in negative SLNs between the researchers could be related to the differences in melanoma thickness and tumor resection/SLN interval [19,20]. Specifically, patients with larger Breslow thickness (>1.5 mm) and shorter intervals (<44 days) appear to present less mature and functional LCs, in line with reports suggesting that primary melanoma immunologic conditioning of the SLNs may sustain the metastatic spread [47,48]. Additional evidence was described by Romoli et al. [15], correlating LC phenotype and melanoma Breslow’s thickness. The Authors studied the APM component expression depending on melanoma Breslow’s thickness: SLN LCs APM level was lower in patients with a thick Breslow, suggesting a melanoma-induced LCs functional deficiency related to the melanoma aggressiveness.

### 4.4. The Enzyme Indoleamine 2,3-Dioxygenase (IDO)

IDO enzymes (IDO1 and 2), responsible for Tryptophan (TRP) degradation and Kynurenines (KYN) production, are key factors in the tolerance process, immune escape mechanisms and tumor-induced immunosuppression [8,49]. Studies on melanoma have highlighted that IDO enzymes, by inducing FOXP3 release in the tumor microenvironment, promote effector T-cell suppression and generation of regulatory T cells (Tregs) [49]. Consistently, a significant increase in circulating Tregs occurs in the peripheral blood of patients with advanced melanoma and positive SLNs, possibly influencing host immune response [50,51,52,53]. Given the potential interactions, the relation between melanoma cells, DC/LCs and IDO enzymes is a topic of interest, and the available literature is discussed as follows.

#### 4.4.1. Dendritic Cells and the Enzyme Indoleamine 2,3-Dioxygenase (IDO)

In the SLN of patients with melanoma, IDO1 expression has been described on DCs [53]. Specifically, IDO1+ DCs are frequent in metastatic SLNs, correlating with a worse clinical prognosis [53]. Accordingly, IDO1 expression in SLN currently represents a negative prognostic factor [54], and IDO1 inhibitors are a focus of research, potentially reverting tumor tolerance [9,55,56].

#### 4.4.2. Melanoma Cells and the Enzyme Indoleamine 2,3-Dioxygenase (IDO)

Primary and metastatic melanoma cells express IDO1 [57,58], and its expression is promoted by in vitro stimulation with interferon-gamma [57]. A correlation between IDO1 expression in melanoma cells and poor prognosis has been demonstrated [57]. Indeed, IDO1 levels are higher in primary and metastatic LNs of patients with poor survival compared to those with long survival, in which IDO1 is poorly detected or absent [57]. Furthermore, IDO1 expression has been correlated with the Breslow thickness of the primary cutaneous melanoma [57]. Overall, IDO1 expression in both APCs and melanoma cells correlates with poor prognosis [58], and IDO enzymes appear to have an influence on the immune system in terms of recognizing melanoma cells and setting up an early response, thus promoting tumor immune evasion and cancer spread.

#### 4.4.3. Langerhans Cells and the Enzyme Indoleamine 2,3-Dioxygenase (IDO)

Under physiological cutaneous conditions, IDO1 expression does not occur in immature human LCs. Contrariwise, in vivo studies reported that a fraction of mature CD83+ LCs expressed IDO following induced maturation, suggesting potential regulatory/inhibitory functions [7,9,52]. Considering that DCs and LCs, in particular, are promising targets for future immunotherapeutic strategies [4,11], IDO and its correlation with CD83 have been widely investigated in SLN LCs of melanoma patients.

Gerlini et al. [13] studied the tolerogenic enzyme IDO1 in melanoma SLN LCs, revealing IDO1 expression in a small percentage of LCs located in the T cell-rich area of SLNs. At flow cytometry analyses, IDO1 expression was documented in both melanoma-positive and melanoma-negative SLN LCs [13]. However, a higher (but not significative) co-expression of Langerin+ LCs and IDO enzyme was described in positive SLNs (4,18%) versus 3.63% at the negative ones. Furthermore, positive SLNs hosted lower levels of mature CD83+ LCs compared to melanoma-negative SLNs (40.51%), confirming previous reports [20,21]. Regarding morphology, SLN LCs presented thin and long dendrites, which is the typical healthy dendritic appearance, contrasting previous studies reporting morphological alterations of DCs within melanoma SLN [28,33].

**Figure 2:**
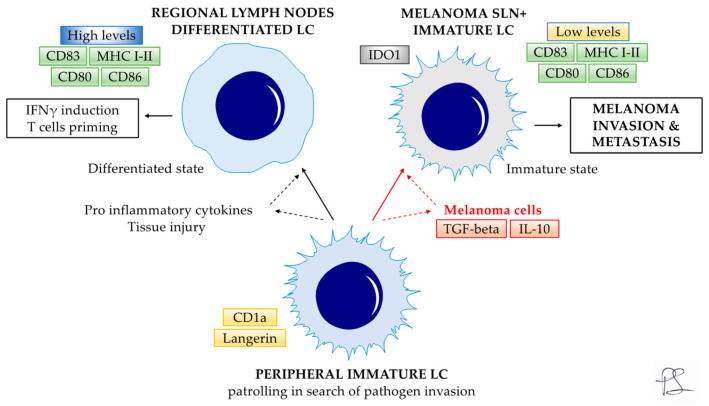
Peripheral resident LCs are immature (CD1a, Langerin). In response to tissue injuries, they migrate to regional lymph nodes, reaching a full maturation state (High levels of MHC I-II, CD80/CD86, and CD83), becoming effective antigen-presenting cells able to induce anti-cancer response. Melanoma cells’ tolerogenic mechanisms (TGF-beta and IL-10) promote functional deficits in SLN LCs, inducing an immature immunophenotype (Low levels of MHC I-II, CD80/CD86, and CD83), affecting their immune-regulatory functions (IDO1), possibly promoting tumor tolerance. Solid black and red lines indicate the LC behavior. Dashed red and black lines indicate a positive stimulus. LC—Langerhans cells; SLN—Sentinel lymph node.

In order to better understand SLN LCs functional properties, the Authors focused on LCs IDO1 and CD83 relative expression in both melanoma-positive and negative SLNs. Interestingly, they identified four specific SLN LCs subsets at double IDO1/CD83 staining. The groups included real mature LCs (IDO1-, CD83+); real immature LCs (IDO1-, CD83-) which are phenotypically similar to peripheral immature LCS [52]; tolerogenic mature LCs (IDO1+, CD83+), exerting regulatory functions and possibly involved in T-cell tolerance [26,52]; and, finally, tolerogenic immature LCs (IDO1+, CD83-). Of note, the first three subsets were not correlated to significant differences. By contrast, the tolerogenic immature IDO1+CD83- LCs were significantly increased in positive SLNs. Consequently, tolerogenic immature IDO1+CD83-LCs could be involved in LNs metastasis arrival/homing and melanoma proliferation. Additionally, melanoma-induced IDO1 expression and CD83 downregulation could represent a new tumor escape mechanism. The authors also correlated the different SLN LC subset frequencies with primary melanoma Breslow’s thickness and mitotic rate (MR). No significative differences were found for Breslow’s thickness. However, a direct correlation was described for tolerogenic immature SLN LCs frequency and the primary melanoma MR rates: 3.25% vs. 10.52% vs. 19.31% for low MR (<1), intermediate MR (>1 and <5), and high MR (≥5), respectively. Based on the aforementioned findings, melanoma cells could induce a transient immune response, increasing inflammatory cytokines levels, inducing CD83 and IDO1 expression in LCs, generating CD83+ LCs, either IDO1+/−, with correlation to primary melanoma MR rates. Then, CD83+ LCs, IDO1+/− (real mature SLN LCs and regulatory/inhibitory SLN LCs) migrate to SLN, possibly modulating the escape mechanism in melanoma (Figure 2).

#### 4.4.4. Melanoma-Induced IDO-KYN-AhR Interactions on Langerhans Cells Promote a Vicious Cycle Sustaining Immune Tolerance

Additional evidence has highlighted that following disease progression, a subset of melanoma cells might also express IDO1 [58]. Moreover, functional Aryl hydrocarbon receptor (AhR) is expressed in human skin LCs [16]. AhR is an endobiotic receptor that represents the KYN target, which mediates the main IDO1 suppressive actions [16]. KYN-induced AhR stimulation has been associated with IDO1 increased expression in LCs without promoting CD83 expression [16]. Consequently, increased IDO1 expression in both APCs and melanoma cells could promote KYN-AhR interactions, stimulating peripheral LC and leading to a vicious cycle with further LCs IDO1 expression, but without improving CD83 expression. Thus, immunosuppressive tumor cytokines and AhR stimulation promote CD83 downregulation and generation of CD83- IDO1+LCs (immature tolerogenic LCs) and/or CD83- IDO1-LCs (real immature LCs). These are ineffective in stimulating T cells. This hypothesis might be consistent with the frequencies detected for the four SLN LCs subsets in melanoma patients, being CD83+IDO1- and CD83-IDO1- the main SLN LCs subsets, and CD83+IDO1+ SLN LCs, the lower one, slightly affected by melanoma. On the contrary, CD83-IDO1+ SLN LCs were highly affected by melanoma, being significantly higher in primary melanoma with higher MR number and melanoma-positive SLNs. Notably, TRP depletion and KYN production may be promoted by SLN IDO1+ DC subsets, including pDCs, dDCs and LCs, leading to effector T cells impairment, enhancing Tregs, sustaining a tolerogenic microenvironment, promoting SLN melanoma metastases.

### 4.5. Sentinel Lymph Node Langerhans Cells Are Not Irreversibly Impaired by Melanoma Cells

A very important aspect is that stimulating immature SLN LCs by appropriate stimuli can influence their functionality and maturation state. In particular, Gerlini et al. [13] studied ex vivo to see if melanoma SLN LCs could still achieve a mature phenotype (CD83 higher levels). At fluorescent immunocytochemistry and flow cytometry analysis, they demonstrated that strong CD83 up-regulation occurs following appropriate inflammatory cytokines stimulation. Furthermore, some LCs re-expressed cytoplasmic Langerin, an additional maturation marker. These markers are central to LCs-mediated T-cell activation, proliferation, and immune control [21]. In addition, van den Hout et al. and Sluijter et al. [59,60] revealed that peri-tumoral injection of Granulocyte/Macrophage-Colony Stimulating Factor (GM-CSF) before SLN biopsy reduces SLN immunosuppression. Specifically, they reported a pilot Phase II clinical trial including 28 clinical-stage I-II melanoma patients receiving, before the SLN biopsy, intradermal injections of saline or DC-targeting agents (low-dose Cytosine-phosphate-Guanine (CpG-B), alone or combined with GM-CSF) around the melanoma excision site [59,60]. In their pilot study, local low-dose CpG administration, irrespective of GM-CSF co-administration, resulted in increased frequencies of melanoma-specific CD8(+) T cells and possible recruitment of effector NK cells, potentially re-activating an antitumor activity at the SLNs. Their study represents solid evidence of the feasibility of a DC-based anti-melanoma vaccine. These findings suggest that melanoma cells do not irreversibly impair SLN LCs and their physiological T-cells’ regulatory (inhibitory/stimulatory) function, potentially representing a target for cancer vaccine, paving the way for new immunotherapeutic approaches [61,62]. Future research will have to prove its effectiveness.

### 4.6. Langerhans Cells and Immunotherapy: Future Directions

APCs, particularly LCs, perform immunoregulatory functions at the skin level, interacting with both exogenous and skin tumor antigens [4,11]. Numerous reports have described immuno-phenotypic and functional defects of SLN DCs in cancer patients, particularly in melanoma [10,20,21,63,64]. The CD83 molecule, a marker of DC mature immunophenotype, is poorly expressed by SLN LCs of melanoma patients, possibly explaining their inability to stimulate anti-melanoma immunity [20,21].

In vitro experiments have highlighted that CD83 expression can be restored, with the hope of developing cancer vaccines capable of pharmacologically reactivating DCs/LCs and, consequentially, T cells, restoring antitumor immunity [13,21]. In addition, Gerlini et al. [18] showed in vivo that electrochemotherapy can promote LC activation, migration to regional lymph nodes and maturation (CD83 expression), suggesting potential combination treatments. Furthermore, primary melanoma cells and peri-tumoral APCs express IDO enzymes, the main ones responsible for immune tolerance, contributing to tumor escape mechanisms [5,56,58].

Recently, IDO1 inhibitors have been included in clinical trials in combination with chemotherapy and immunotherapy, such as anti-CTLA-4 and/or PD-1, to restore anti-melanoma immunity [9,55,56]. These could act as immune adjuvants, reprogramming the appropriate immune response [55,56]. Moreover, Tajpara et al. [14] studied the ex vivo expression and activation of sensing pattern recognition receptors in LCs as possible molecular targets for therapeutic vaccination. Of note, LCs expressed melanoma differentiation-associated protein 5 (MDA5) [14]. MDA5 stimulation with specific ligands induced IFN-α2, IFN-β, TNF-α, IL-6 and IL-8 cytokines levels, representing a potential target for epi-cutaneous delivery of therapeutic vaccines [14].

Ex vivo-generated autologous monocyte-derived or CD34+ hematopoietic precursor-derived DC are the main sources employed in DC-based immunotherapies [24,39]. Based on in vivo animal trials, DC vaccination has been occasionally related to clinical benefits, but the efficacy is still limited [65]. Indeed, researchers are still testing several DC subsets, stimulation techniques and ways of administration to obtain a safe and effective vaccine [17,22,66,67,68]. Additional research is expected towards new effective DC/LC-directed cancer vaccines.

## 5. Conclusions

**Figure 3:**
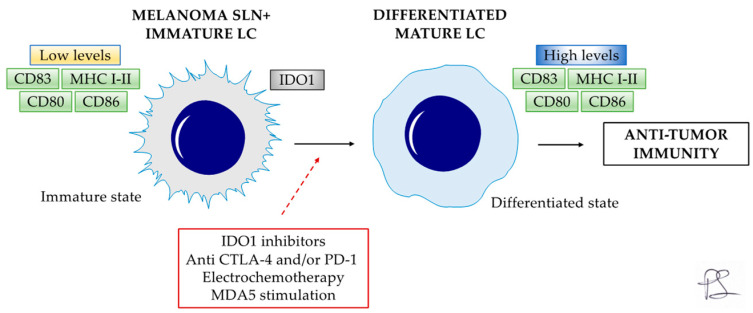
Appropriate stimuli (IDO1 inhibitors, anti-CTLA-4 and/or PD-1, electrochemotherapy, and MDA5 stimulation) could restore LC functions (High levels of MHC I-II, CD80/ CD86, and CD83), paving the way for future immunotherapeutic strategies and future cancer vaccines capable to pharmacologically reactivate DCs/LCs and restore antitumor immunity. Solid black lines indicate the LC behavior. Dashed red line indicates a positive stimulus. LC—Langerhans cells; SLN—Sentinel lymph node.

The present systematic literature review focused on LC in melanoma based on personal authors’ experience and the best available evidence. From the present review, DCs and LCs are involved in immunoregulatory functions promoting T-cell activity, including tumoral control. SLN LCs in patients with melanoma are functionally defective (low CD80/86 co-stimulatory molecules), immature (low CD83), and express IDO enzymes. The latter are key immune escape mechanisms that promote an immunosuppressed environment at the SLN level and favor LNs’ metastasis arrival/homing and melanoma proliferation. However, LCs are not irreversibly impaired or morphologically defective due to melanoma. Indeed, appropriate stimuli such as IDO1 inhibitors, anti-CTLA-4 and/or PD-1, electrochemotherapy and MDA5 stimulation seem to restore LC functions, paving the way for future immunotherapeutic strategies and future cancer vaccines capable of pharmacologically reactivating DCs/LCs and restore antitumor immunity (Figure 3). It is hoped that based on the considerations of this article, future research will be dedicated to LC cells and their promising role in melanoma.

## Figures and Tables

**Table 1:** Clinical evidence for the role of LCs in melanoma.

Reference	Article Type	Objective of the Study	Results
Gerlini et al., 2022 [13]	Research article	To investigate SLN LCs characteristics in patients with melanoma by immunohistochemical staining for IDO1 (a marker for immunotolerance)/CD83 (a marker for maturation profile).	Metastatic SLNs presented a statistically significant increased subset of tolerogenic immature LC (IDO1+CD83−) compared to non-metastatic SLNs.
Tajpara et al., 2018 [14]	Research article	To study the expression and activation of sensing pattern recognition receptors in LCs and keratinocytes of human skin as possible molecular targets for therapeutic vaccination.	LCs exclusively expressed melanoma differentiation-associated protein 5 (MDA5), which could represent a target for the epicutaneous delivery of therapeutic vaccines.
Romoli et al., 2017 [15]	Research article	To evaluate HLA-class I APM components in LCs.	APM levels were low in immature epidermal LCs compared to mature LCs. Moreover, the APM component was significantly lower in SLN LCs of patients with thick melanomas compared to thin/intermediate lesions.
Koch et al., 2017 [16]	Research article	To evaluate the expression of an immune regulator in LCs: the AhR and the functional consequences.	AhR activation upregulates IDO expression in LCs, possibly dampening allergen-induced inflammation in atopic dermatitis.
Chung et al., 2017 [17]	Phase I vaccine trial	To evaluate the effectiveness of a vaccine based on autologous LCs in melanoma patients.	LCs were electroporated with murine tyrosinase-related peptide-2 (mTRP2) mRNA. The vaccines were safe and immunogenic in 9 patients.
Gerlini et al., 2013 [18]	Research article	To assess the presence of DCs in the inflammatory perilesional infiltrate following electrochemotherapy.	Electrochemotherapy induced LC activation, migration to regional lymph nodes and maturation (CD83 expression).
Van den Hout et al., 2012 [19]	Letter to the editor (response to Gerlini et al., 2012 [20])	To characterize human DC subsets residing in SLNs of melanoma patients.	Melanoma-draining SLNs host skin-derived cDCs (in particular LCs), and the maturation state depends on Breslow thickness and the excision interval state.
Gerlini et al., 2012 [20]	Letter to the editor (response to Van de Ven et al., 2011 [21])	To characterize human DC subsets residing in SLNs of melanoma patients.	Melanoma-draining SLNs host immature skin-derived cDCs (in particular LCs), thus presenting an inferior ability to activate T-cells.
Van de Ven et al., 2011 [21]	Research article	To characterize human DC subsets residing in SLNs of melanoma patients.	Melanoma-draining SLNs host skin-derived cDCs (in particular LCs) that are characterized by a high maturation state but an inferior ability to activate T-cells.
Romano et al., 2011 [22]	Phase I clinical trial	To compare LCs in vivo tumor immunogen activity with monocyte-derived DCs (moDCs) in patients with AJCC stage III or IV melanoma.	The two vaccines were both safe and immunogenic, but LCs synthesized much more IL15 than moDCs.
Stoitzner et al., 2008 [4]	Research article	To evaluate the role of LCs in tumor immunotherapy following epicutaneous immunization with OVA protein (directed against OVA-expressing melanoma cells).	LCs play a central role. Indeed, in a mouse model, LC depletion reduced the tumor-protective effect.
Cao et al., 2007 [23]	Research article	To assess DCs ability to present melanoma antigens to CD8+ T cells.	In vitro, both LCs and DCs can cross-present melanoma antigens to naive CD8+ T cells, promoting host immune response.
Fay et al., 2006 [24]	Phase I clinical trial	To evaluate the potential role of DCs vaccine (including LCs) loaded with melanoma antigens.	The vaccine was safe and immunogenic in 10 patients.
Bennaceur et al., 2006 [25]	Research article	To assess the effect of human melanoma cells purified gangliosides on LCs.	In vitro, GM3 and GD3 gangliosides impaired LC maturation, reducing their T immune-stimulating effect.
Von Bubnoff et al., 2004 [26]	Research article	To study LC functions.	LC stimulation with interferon-c induces IDO1 expression, inhibiting T-cell proliferation.

## Data Availability

The original contributions presented in the study are included in the article; further inquiries can be directed to the corresponding author/s.
